# A double blind, placebo-controlled, randomized clinical trial that breast milk derived-*Lactobacillus gasseri* BNR17 mitigated diarrhea-dominant irritable bowel syndrome

**DOI:** 10.3164/jcbn.17-73

**Published:** 2018-01-11

**Authors:** Suk Pyo Shin, Yoon Mi Choi, Won Hee Kim, Sung Pyo Hong, Jong-Min Park, Joohee Kim, Oran Kwon, Eun Hyun Lee, Ki Baik Hahm

**Affiliations:** 1 Department of Gastroenterology, Hallym University Sacred Hospital, Chooncheon, Korea; 2 Digestive Disease Center, CHA University Bundang Medical Center, 59 Yatap-roBundang-gu, Seongnam 13496, Korea; 3 CHA Bio Complex CHA Cancer Prevention Research Center, Pangyo, 13488, Korea; 4 Department of Nutritional Science and Food management, Ewha Woman University, 52 Ewhayeodae-gil, Seodaemun-gu, Seoul 03760, Korea; 5 Graduate School of Public Health, Ajou University, Suwon, 16499, Korea

**Keywords:** diarrhea-dominant IBS, *Lactobacillus gasseri* BNR17, IBS-QOL, colon transit time, microbiota

## Abstract

The exact pathogenesis of diarrhea-dominant irritable bowel syndrome (IBS) is not known, but the abnormal microbiota of the gastrointestinal tract is considered to be one of the important contributing factors as in other gastrointestinal diseases such as inflammatory bowel disease, antibiotic-associated diarrhea, and colorectal cancer as well as systemic diseases. Though diverse trials of probiotics had been continued in the treatment of diarrhea-IBS, only a few proved by randomized clinical trial. To prove the efficacy of *Lactobacillus gasseri* BNR17 isolated from breast milk in patients with diarrhea-IBS, prospective, randomized, placebo controlled clinical trial was done including health related-quality of life analysis, colon transit time, and the changes of fecal microbiota. BNR17 significantly improved the symptoms of diarrhea compared to control group. Health related-QOL analysis showed significant improvement of abdominal pain, distension, disturbed daily life, and mean defecation frequency with BNR17. On comparative CTT before and after BNR17, 6 out of 24 subjects showed significant correction of rapid colon transit pattern, while only 2 out of 24 in placebo (*p*<0.01). Upon fecal microbiota analysis, BNR17 significantly increased *B. fecalis*, *E. rectale*, *C. aerofaciens*, *F. prausnitzil* and *B. steroris*. Conclusively, *Lactobacillus gasseri* BNR17 can be a potential probiotics to ameliorate diarrhea-IBS.

## Introduction

Probiotics have been extensively studied over the past several years in the treatment of diverse diarrheal diseases.^(1)^ However, in spite that most randomized controlled trials and subsequent meta-analyses suggest benefit for probiotics in the treatment or prevention of irritable bowel syndrome (IBS), antibiotic-associated diarrhea,^(2)^ inflammatory bowel disease (IBD),^(3)^ the real clinical efficacy is still inconclusive. Therefore, the efforts to develop effectiveprobioticlactic acid bacilli (LAB) strain are actively on-going since reported benefits were different depending on strain.^(4,5)^

IBS patients are known to have a substantially reduced health related-quality of life (HR-QOL),^(6)^ by which the improvement of IBS-QOL reflects much better than the subjective changes of patients symptoms and visual analogue changes.^(7,8)^ Rapid colon transit time (CTT) has been acknowledged as basis for diarrhea symptom in patients with IBS^(9)^ possibly through increased intestinal permeability,^(10)^ increased visceral hypersensitivity and lowered water resorption.^(11)^ Also, an “imbalance of the microbiota”, a dysbiosis, has been associated with different GI diseases including *Helicobacter pylori*-associated gastritis, IBD, IBS, some infectious intestinal diseases such as *Clostridium difficile* colitis, and even colon carcinogenesis.^(12,13)^ Until now, the trials regarding the efficacy assessment of probiotics were mostly based on the changes of symptoms by visual analogue scale (VAS) or other subjective symptom changes, after which the results of Cochrane meta-analysis did not show significant impact.

*Lactobacillus gasseri* (*L. gasseri*), in the genus of Lactobacillus acidophilus, is the most well-known probiotics and one of the most critical constituents of gut flora.^(14)^ *L. gasseri* BNR17 was isolated from breast milk collected from healthy lactating females within two weeks of parturition in our author’s laboratory. During the study that *L. gasseri* BNR17 showed utmost efficacy on obesity, we have noticed breast milk derived BNR17 might improve bowel function,^(15,16)^ in the current study, we evaluated the efficacy of probiotic *L. gasseri* BNR17 in subjects with diarrhea-dominant IBS incorporating specific IBS-QOL scale, the changes of CTT and blood chemistry, and the fecal microbiota along with the changes of diarrhea symptoms.

## Methods

### Study design

A single center, randomized, double-blind and placebo-controlled clinical trial was carried out to examine the efficacy and safety of *L. gasseri* BNR17 in patients with diarrhea dominant IBS. Treatment duration was 8 weeks with 2 follow-up visits of 4 week intervals. This trial was approved by the Institutional Review Board in CHA Bundang Medical Center, Seongnam, Korea (BD #2013-064) and registered in the International Clinical Trials Registry Platform of WHO with the following identification number of KCT0000969. All volunteers gave written informed consent prior to participation in this trial. Subjects between 20–55 years of age with diarrhea dominant IBS according to the Rome III criteria were eligible for inclusion. Subjects were excluded if they took any medication for IBS, previous intake of probiotics, antibiotics or anti-inflammatory drugs within 4 weeks before first visit. Subjects who had liver, kidney or psychiatric disorder were also excluded and pregnant or breast feeding women, alcoholics were also excluded. Compliance was calculated as the percentage of planned ingestion of the product and subjects at least 80% of compliance were analyzed. As randomization, subjects received number according to the registration order and assignment was performed using computer generated random list. The subjects and investigators remained blind until completion of this trial.

### Study products

The test product contained *L. gasseri* BNR17 (250 mg per capsule, Bioneer Inc., Daejeon, Korea), maltodextrin (6 mg), microcrystalline cellulose (35%) and magnesium stearate (1%). Placebo capsules were identical in all aspects, but maltodextrin was the substitution of *L. gasseri* BNR17. All subjects were instructed to take 4 capsules per day (2 capsules after breakfast and dinner) and subjects in test group could take 10^10^ CFU/day of *L. gasseri* BNR17.

### Assessments and study endpoints

The primary endpoint of this study was the improvement of the bowel function. Secondary endpoints included the improvement on blood glucose control and safety of this product. Bowel function was assessed by IBS-QOL questionnaire, Questionnaire for health status, Questionnaire for the degree of IBS symptom, Subjective global assessment of IBS symptom improvement, Bristol stool scale at week 0, 4 and 8, colon transit time at week 0 and 8 and pyrosequencing in feces at week 0 and 8. Colon transit time was calculated with 4-day method. Subjects took a capsule which contains radiopaque markers (Kolomark^®^) a day for 3 days and followed by abdominal X-ray on day 4. Pyrosequencing study of fecal microbiota was performed only in participants, whose body mass index (BMI) was over 25/m^2^ sent with written consent. Safety was assessed with vital sign on every visit and blood laboratory test such as CBC c diff., AST, ALT, BUN, creatinine, uric acid, total protein, albumin and urinalysis (blood, protein, glucose, pH). Serum glucose, insulin and HbA1c at week 0 and 8 were also analyzed to assess the effect of *L. gasseri* BNR17 on blood glucose control.

### Statistics

Per-protocol analysis, which included the participants who consumed at least 80% of the study material provided, was performed. Variables were tested for normal distribution using the Shapiro-Wilk test. Normally distributed variables were analyzed using the Student’s *t* test for the between-group comparisons and the paired *t* test for the within-group comparisons. Non-normally distributed variables were analyzed using the Wilcoxon rank sum test for the between-group comparisons and the Wilcoxon signed rank test for the within-group comparisons. Within-group comparisons were performed between week 0 and week 8. Categorical variables were analyzed using Chi-square test and Fisher’s exact test. All statistical analyses were performed using the SAS program package ver. 9.3 (SAS Institute, Cary, NC). A two-tailed value of *p*<0.05 was considered to be significantly different.

## Results

### RCT of *L. gasseri* BNR17 in diarrhea-dominant IBS

60 subjects were included and were divided into two groups. 30 subjects were assigned to take *L. gasseri* BNR17 group and other 30 subjects to take placebo through randomization assignment. Onto per protocol analysis (PP), in *L. gasseri* BNR17 group, 2 subjects were discontinued intervention and 4 subjects did not achieve medication adherence rate at 80%, after which 24 subjects were included in the analysis. In placebo group, 2 subjects were discontinued intervention and 1 subject did not achieve medication adherence rate at 80%, after which 27 subjects were included in the analysis. As seen in Table 1, there were no significant differences in the sex ratio, age, body weight, BMI, systolic blood pressure (SBP), diastolic blood pressure (DBP) and smoking between placebo and BNR17 group. However, *L. gasseri* BNR17 group had significantly higher drinker than placebo group (*p*<0.05). As results of RCT, according to the changes of visual analogue scale (0–5 scale), especially the diarrhea symptoms were improved in 7 of 27 (25.9% in placebo group) and in 16 of 24 (66.7% in *L. gasseri* BNR17 group), significant difference between group (*p*<0.01).

### Comparative changes of IBS-QOL questionnaire in diarrhea-dominant IBS

**Fig. 1:**
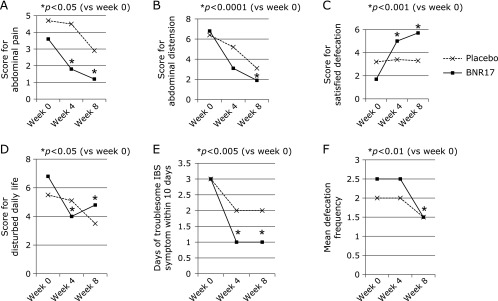
The changes of IBS-QOL questionnaire after placebo or *L. gasseri* BNR17, symptom changes. Among IBS-QOL scale, statistically significant changes in score after *L. gasseri* BNR17 were as follows; the changes of abdominal pain (*p*<0.05), abdominal distension (*p*<0.0001), satisfied defecation (*p*<0.001), disturbed daily life (*p*<0.05), the days of troublesome IBS symptoms (*p*<0.005), and mean defecation frequency (*p*<0.01). Detailed changes in score were presented in Table 2.

**Fig. 2:**
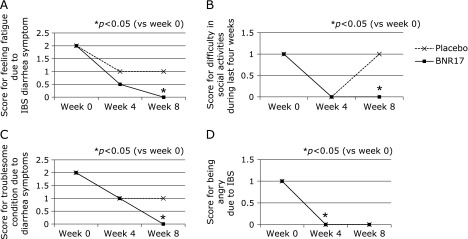
The changes of IBS-QOL questionnaire after placebo or *L. gasseri* BNR17, life style changes. Among IBS-QOL scale, significant changes among IBS-QOL were as follows; feels fatigue due to IBD diarrhea symptom (*p*<0.05), difficulty in social activities last four weeks (*p*<0.05), troublesome and not stable due to diarrhea symptoms (*p*<0.05), exhausted due to IBS symptoms (*p*<0.05), angry due to irritable symptoms of IBS (*p*<0.05), and restricted and limited in familial activities (*p*<0.05). Detailed changes in score were presented in Table 3.

IBS-QOL questionnaire was specifically developed for the current study.^(17)^ In 24 questionnaires out of 29 questionnaires, the both groups showed improvement of symptoms during 8 weeks, signifying that the diverse symptoms concurring in diarrhea-dominant IBS can be improved with placebo. However, as shown in Table 2 and Fig. 1, the mean score for abdominal pain, abdominal distension, satisfied defecation and disturbed daily life and changes of days of troublesome IBS symptoms within 10 days were statistically significantly reduced in *L. gasseri* BNR17 group compared to placebo group (*p*<0.05). In additional questionnaires shown in Table 3 and Fig. 2, *L. gasseri* BNR17 group showed significant improved scores compared to placebo group at 8 week in generalized symptoms like fatigue due to IBD diarrhea symptom (*p*<0.05), difficulty in social activities last four weeks (*p*<0.05), troublesome diarrhea symptoms (*p*<0.05), exhausted due to IBS symptoms (*p*<0.05), angry due to irritable symptoms of IBS (*p*<0.05), and restricted and limited in familial activities (*p*<0.05).

### Subjective global assessment of IBS symptom improvement

The number of subjects who responded that have improved or not improved symptom are shown in Table 4. The number of subjects with symptom improved was larger in *L. gasseri* BNR17group than placebo group, but it was not significant statistically.

### The changes of metabolic parameters including glucose, HbA1c, and insulin

Since the preliminary study showed that *L. gasseri* BNR17 improved metabolic syndrome, in this study,^(14–16)^ we had measured the changes of glucose, HbA1c, and serum level of insulin according group. As seen in Table 5, the mean fasting blood glucose was significantly decreased during 8 weeks in *L. gasseri* BNR17 group (101.5 mg/dl at week 0 and 97.5 mg/dl at week 8, *p*<0.05) but not in placebo group. However, HbA1c and insulin level did not show significant changes in any group. Since the most subjects were without diabetes, these changes only suggest the possible application for metabolic advantage in patients with diabetes.

### The changes of colonic transit time (CTT)

**Fig. 3:**
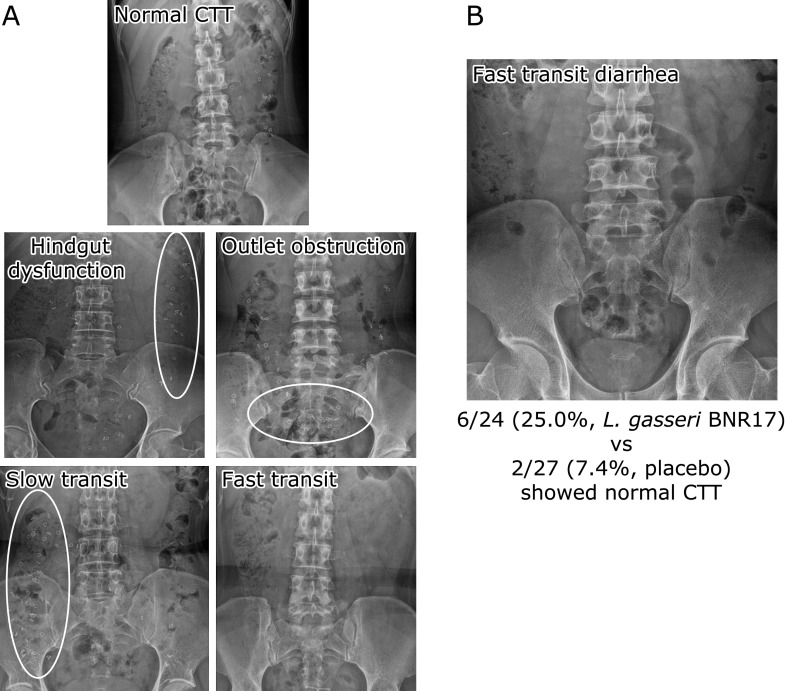
CTT pattern and the changes of CTT with *L. gasseri* BNR17. (A) CTT patterns, hindgut dysfunction, oulet obstruction, slow transit, fast transit (B) CTT changes from fasting transit diarrhea to normal pattern after *L. gasseri* BNR17 in 6 subjects with diarrhea-dominant IBS.

The CTT changes according to group are shown in Table 5. The mean CTT was 5.4 h at week 0 and 19.2 h at week 8 in subjects with diarrhea dominant IBS treated with BNR17 and 8.4 h week 0 and 13.2 h at week 8 in placebo treated group. CTT was significantly increased during 8 weeks in *L. gasseri* BNR17 group but not in placebo group (*p*<0.05). Various types of colonic transit type were found in this study and typical cases are shown in Fig. 3A. The numbers of subject who changed from fast transit time to normal transit time are 2 out of placebo group, but was noted in 6 out of *L. gasseri* BNR17 group (*p*<0.01, Fig. 3B).

### Changes of fecalmicrobiota according to group

**Fig. 4:**
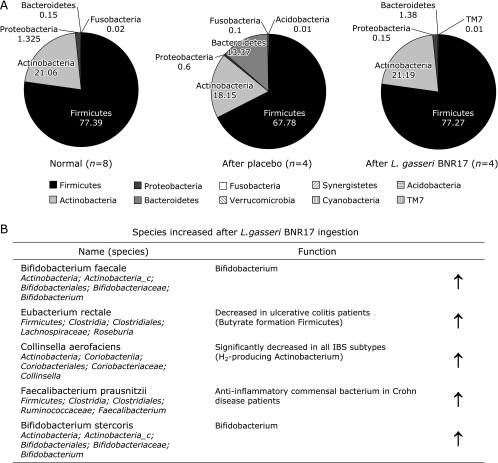
Microbiota changes. (A) phylum levels. (B) Genus level. Significant increments in *Bifidobacteriumfaecalis*, *Eubacteriumrectale*, *CollinsellaAerofaciens*, *Faecalibacteriumprausnitzii*, and *Bifidobacteriumstercoris* were noted with *L. gasseri* BNR17 as part of improved symptom of diarrhea-dominant IBS.

 Among 51 subjects, 8 subjects who presented with BMI >25/m^2^ were included in these analysis of fecal microbiota as written in inclusion criteria. On phylum assessment, subjects receiving *L. gasseri* BNR17 showed significant increment in *Actinobacteria* (8.1% to 21.19%), decrement in *Proteobacteria* (2.2% to 0.15%). On placebo administered cases, no significant changes were noted except significant increment of *Bacteroidetes*. On further Genus analysis, significant changes were prominently noted in BNR17 administration that increment in *Lactobacillus* and *Bifidobacterium*, whereas decrement in *Blautia* and fecalibacterium (Fig. 4). Significantly increasing species were described in Fig. 4B, all of these microbiome changes relevant to *L. gasseri* BNR17 were, we inferred, compatible with afore-mentioned improvement of diarrhea dominant IBS.

### Safety assessment

Vital sign, CBC with differentiation, biochemical changes including AST, ALT, BUN, creatinine, uric acid, total protein and albumin, and urinalysis were not changed significantly in the two groups (data not shown).

## Discussion

From the current a randomized, double blinded, prospective, comparative clinical trial, we found that 8-week ingestions of breast milk derived *L. gasseri* BNR17 significantly improved the IBS-QOL in subjects with diarrhea-dominant IBS accompanied with healthymicrobiota changes, improved colon transit in some. Thoughmeta-analyses that have evaluated the efficacy of probiotics in physiological and pathological conditions such as antibiotic or chemotherapy-associated diarrhea, *Clostridium difficile*-associated pseudomembranous colitis, IBS, constipation, inflammatory bowel disease, respiratory tract infection, ventilator-associated pneumonia, non-alcoholic fatty liver disease, periodontitis, vaginosis, urinary tract infections, pancreatitis, various hospital infection, necrotisingenterocolitis in premature infants,^(5,18)^ the results are contradictory except antibiotic- and *Clostridium difficile*-associated diarrhea. After the current status, we can add evidence that *L. gasseri* BNR17 can be effective for diarrhea-dominant IBS.

Though there had been many clinical trials to investigate the effect of probiotics in patients with IBS, most trials were open-labeled proof of the effectivenessof probiotics compared to placeboin IBS symptoms,^(19–22)^ only some were blinded trial, but mostly focused on visual analogue scaled symptom changes and some more parameters.^(23–28)^ Since a meta-analysis of RCT of IBS patient showed that the placebo response rate ranged from 16.0 to 71.4% in assessed global response,^(29)^ the placebo effect is important in functional disorder and psychological factors also affect significant levels of IBS symptoms.^(30)^ In our trial, more than 80% of IBS-QOL were improved with placebo trial, both *L. gasseri* BNR17 and placebo improved symptoms of subjects with IBS in some points of QOL. However, we could clearly show significant beneficiary effect of *L. gasseri* BNR17 onto diarrhea-dominant IBD including the following analysis that statistically significant in some IBS-QOL analysis, improved CTT, and enrichments of gut microbiota relevant to patient symptoms.

Regarding the implication of CTT, some studies showed accelerated CTT in diarrhea-dominant IBS patients,^(31)^ whereas not in other studies.^(32,33)^ In studies performed with Korean population, the mean CTT was 20 to 30 h in asymptomatic normal subjects,^(34)^ but in this current study, average CTT of all subjects was 7.0 h, which was considerably accelerated compared to average transit time of normal Korean population, a part of pathogenetic mechanism explaining of diarrhea-dominant IBS. Similar to previous studies,^(35)^ *L. gasseri* BNR17 ingestion prolonged CTT to the near average level of Korean population. Because of disturbances of colonic motility play an important role in diarrhea symptom of IBS, the effect of *L. gasseri* BNR17 on the colon motility is very contributoryto IBS and imposes a potential treatment option to subjects with diarrhea-dominant IBS.

The deranged intestinal microbiota is thought to play important roles in the pathogenesis of IBS since many recent studies demonstrated gut microbial dysbiosis in IBS patients. The putative mechanism show intestinal microbiota orchestrate intestinal homeostasis and function include the alteration of the microbiota-gut-brain axis, quantitative and qualitative changes in the microbiota, activation of mucosal immunity and inflammation, altered mucosal permeability, and the epithelial barrier and sensory-motor disturbances.^(36)^ In this presented study, *Firmicutes* took the most microbiome at the phylum level in the both groups of subjects with IBS before intake *L. gasseri* BNR17. Although we did not compare the microbiome to healthy subjects in whole subjects, the *Firmicutes* levels are high consistent to previous studies which showed the increased ratio of *Firmicutes* to *Bacteroidetes* and reduced quantity of *Actinobacteria* insubjects with IBS.^(37–39)^ The decrease of *Firmicutes* and increase of *Actinobacteria* and *Bacteroidetes* after *L. gasseri* BNR17 ingestion is the outcome suggestive of the correction of the dysbiosis observedin IBS patient. Therefore, we could conclude that *L. gasseri* BNR17 may improve the bowel function in subjects with diarrhea dominant IBS through the correction of the symptomatic related dysbiosis. However, more clear explanation can be possible with more extensive inclusion of subject number as well as elucidations of further mechanistic exploration.

Previous study with *L. gasseri* BNR17 in *db⁄db* mice which have similar characteristics to humans with type 2 diabetes demonstrated the anti-diabetic effect of *L. gasseri* BNR17.^(40)^ We proved the anti-diabetic effect of *L. gasseri* BNR17 in this study, but seem insignificant because included subjects were mostly not diabetic. Although the amount of reduction was only 4 mg/dl during 8 weeks, the difference was significant statistically. The number of subjects whose fasting glucose was above 110 mg/dl was 5 in placebo and *L. gasseri* BNR17 group equally. The mean changes of fasting glucose level in these subgroups were 10.4 mg/dl in *L. gasseri* BNR17 group and 2.8 mg/dl in placebo group. This result implies the possibility that *L. gasseri* BNR17 can be preferred in subjects with DM-related intestinal motility disorder or metabolic syndrome-related IBS. Also, longer study duration and larger amount of subjects with diabetes are needed in this aspect.

*L. gasseri* BNR17 reported not a serious adverse reaction but slightly decrease of hematocrit.^(16)^ In this study, only a slightly decrease of hematocrit was noted without other adverse reactions, but the reduction rated was minimal and the change of hemoglobin was insignificant. Although it is hard to conclude that *L. gasseri* BNR17 inducesanemia, study with larger number of subject with longer duration is needed to clarify it. Another limitation of current study was that we could not explain why the degree of alcohol consumption was different between the two groups, that is, in the *L. gasseri* BNR17 group, subjects took more alcohol, even though assignment was very randomized in clinical trial. The subjects who drink might reduce or stop the alcohol during the study period, which can affect the efficacy of *L. gasseri* BNR17. However, the withdrawal of alcohol increased the CTT,^(41)^ by which no statistical significance of CTT was seen between placebo and *L. gasseri* BNR17. In spite of these limitations, correction of CTT by *L. gasseri* BNR17 was observed in some subjects. Though still the poor understanding of IBS-QOL questionnaire was limitation, since the subtle description regarding questions made participants hard to response, but overall assessment was performed in excellence.

Though more extensive clinical trials are needed with longer duration and larger number of subjects to evaluate the long term effect of BNR17 on bowel function, we could conclude that *L. gasseri* BNR17 derived from human breast milk significantly improved diarrhea-dominant IBS, based on significant microbiota change, bowel function improvement, and fasting glucose control without any serious complication, deserving clinical application in patients with diarrhea-dominant IBS as rescue and ethical medication.

## Figures and Tables

**Table 1:** Baseline characteristics^†^

	Placebo (*n* = 27)	BNR17 (*n* = 24)	*p* value^‡^
Gender (male/female)	10/17	12/12	0.3508
Age (years)	38.0 (30.0, 46.0)	35.0 (32.0, 40.5)	0.2639
Weight (kg)	62.7 (54.0, 77.0)	68.2 (56.4, 82.5)	0.2736
BMI (kg/m^2^)	22.9 (20.1, 27.9)	23.9 (21.6, 27.6)	0.4335
SBP (mmHg)	118.0 (104.0, 134.0)	119.5 (110.0, 135.5)	0.4848
DBP (mmHg)	70.0 (64.0, 77.0)	70.5 (67.0, 81.5)	0.6706
Alcohol consumption (current drinker/former drinker/non-drinker)	15/3/9	22/1/1	0.0066
Smoking (current smoker/former smoker/non-smoker)	2/4/21	5/5/14	0.2714

^†^Median, interquartile range (25%, 75%) in parentheses (all such values). ^‡^Between-group comparisons using Student’s *t* test or Wilcoxon rank sum test for continuous variables and Chi-square test or Fisher’s exact test for categorical variables.

**Table 2:** Questionnaire score changes regarding IBS symptoms^†^

	Placebo (*n* = 27)	BNR17 (*n* = 24)
**Q1. Abdominal pain**		
Week 0 → 4 → 8	4.7 → 4.5 → 2.9	3.6 → 1.8 → 1.2
* p* value	0.1142	0.0032
**Q2. Abdominal distension**		
Week 0 → 4 → 8	6.4 → 5.2 → 3.1	6.8 → 3.1 → 1.9
*p* value	0.1172	<0.0001
**Q3. Satisfied defecation**		
Week 0 → 4 → 8	3.2 → 3.4 → 3.3	1.7 → 5.0 → 5.7
*p* value	0.8426	0.0008
**Q4. Disturbed daily life**		
Week 0 → 4 → 8	5.5 → 5.1 → 3.5	6.8 → 4.0 → 4.8
*p* value	0.1792	0.05
**Q5. Days of troublesome IBS symptoms within 10 days**		
Week 0 → 4 → 8	3.0 → 2.0 → 2.0	3.0 → 1.0 → 1.0
* p* value	0.3424	0.0051
**Q6. Mean defecation frequency**		
Week 0 → 4 → 8	2.0 → 2.0 → 1.5	2.5 → 2.5 → 1.5
*p* value	0.0172	0.0966

^†^Median, interquartile range (25%, 75%) in parentheses (all such values). Q1–Q4: Visual analogue scale, 0 (not at all)–10 (extremely), Q5: day, Q6: number of frequency. *p* value; Within-group comparisons using Wilcoxon signed rank test.

**Table 3:** Diarrhea-IBS QOL assessment [from 0 (not at all) to 4 (extremely)]^†^

**Q8. Feels fatigue due to IBS diarrhea symptom**					**Q6. Difficulty in social activities last four weeks?**			
Week 0	2.0 (1.0, 3.0)	2.0 (1.0 2.5)	0.9604		Week 0	1.0 (0.0, 1.0)	1.0 (0.0, 1.5)	0.9272
Week 4	1.0 (0.0, 1.0)	0.5 (0.0, 1.0)	0.6264		Week 4	0.0 (0.0, 1.0)	0.0 (0.0, 1.0)	0.9584
Week 8	1.0 (1.0, 1.0)	0.0 (0.0, 1.0)	**0.0275**		Week 8	1.0 (0.0, 1.0)	0.0 (0.0, 1.0)	**0.028**
ΔWeek 8–0	−1.0 (−1.0, −1.0)	−1.0 (−2.0, −1.0)	0.2209		Δ Week 8–0	0.0 (−1.0, 0.0)	−0.5 (−1.0, 0.0)	0.2047
*p* value^‡^	<0.0001	<0.0001						
**Q12. Troublesome and not stable due to diarrhea symptoms**					**Q7–9. Exhausted due to IBS symptoms**			
Week 0	2.0 (2.0, 4.0)	2.0 (1.0, 3.0)	0.1826		Week 0	2.0 (2.0, 3.0)	3.0 (3.0, 4.0)	0.0485
Week 4	1.0 (0.0, 10.)	1.0 (0.0, 1.5)	0.8487		Week 4	3.0 (2.0, 3.0)	3.0 (2.0, 4.0)	0.3831
Week 8	1.0 (0.0, 2.0)	0.0 (0.0, 1.0)	**0.0487**		Week 8	3.0 (2.0, 4.0)	3.0 (2.0, 4.0)	0.3913
ΔWeek 8–0	−1.0 (−2.0, 0.0)	−1.5 (−3.0, 0.0)	0.8773		ΔWeek 8–0	0.0 (0.0, 1.0)	0.0 (−1.0, 1.0)	**0.0159**
*p* value^‡^	<0.0001	0.0054			*p* value^‡^	0.0041	0.293	
**Q13. Angry due to irritable symptoms of IBS**					**Q10. Restricted and limited in familial activities?**			
Week 0	1.0 (0.0, 2.0)	1.0 (0.0, 2.0)	0.5648		Week 0	3.0 (2.0, 3.0)	3.0 (2.5, 4.0)	0.3878
Week 4	0.0 (0.0, 1.0)	0.0 (0.0, 0.0)	0.0968		Week 4	3.0 (3.0, 4.0)	3.5 (3.0, 4.0)	0.1214
Week 8	0.0 (0.0, 1.0)	0.0 (0.0, 0.0)	**0.0156**		Week 8	3.0 (3.0, 4.0)	4.0 (3.0, 4.0)	**0.0393**
ΔWeek 8–0	−1.0 (−1.0, 0.0)	−0.5 (−1.5, 0.0)	0.5997		ΔWeek 8–0	1.0 (0.0, 1.0)	0.5 (0.0, 1.0)	0.8218
					*p* value^‡^	0.0176	0.0005	

^†^Median, interquartile range (25%, 75%) in parentheses (all such values). ^‡^Within-group comparisons using Wilcoxon signed rank test.

**Table 4:** Subjective global assessment of IBS symptom improvement

	Placebo (*n* = 27)	BNR17 (*n* = 24)	*p* value^†^
	(improved/not improved)		
Week 0	0/27	0/24	—
Week 4	14/13	15/9	0.4435
Week 8	14/13	18/6	0.0879

^†^Between-group comparisons using Chi-square test.

**Table 5:** The changes of glucose, HbA1c and insulin in fasting blood according to group^†^

	Placebo (*n* = 27)	BNR17 (*n* = 24)	*p* value^§^
Glucose (mg/dl)			
Week 0	99.0 (93.0, 112.0)	101.5 (98.0, 105.5)	0.4499
Week 8	99.0 (92.0, 106.0)	97.5 (94.5, 102.0)	0.6985
ΔWeek 8–0	−2.0 (−5.0, 5.0)	−3.0 (−8.0, 0.5)	0.307
*p* value^‡^	0.4402	0.0138	
HbA1c (%)			
Week 0	5.5 (5.3, 5.8)	5.4 (5.3, 5.8)	0.5184
Week 8	5.5 (5.3, 5.7)	5.5 (5.2, 5.7)	0.5308
Δ Week 8–0	0.0 (−0.1, 0.2)	0.0 (−0.1, 0.1)	0.9772
*p* value^‡^	0.788	0.7894	

^†^Median, interquartile range (25%, 75%) in parentheses (all such values). ^‡^Within-group comparisons using Wilcoxon signed rank test. ^§^Between-group comparisons using Wilcoxon rank sum test.

**Table 6:** The changes of colonic transit time (CTT) according to group^†^ (unit: h)

	Placebo (*n* = 27)	BNR17 (*n* = 24)	*p* value^§^
Week 0	8.4 (2.4, 31.2)	5.4 (0.0, 28.7)	0.3578
Week 8	13.2 (0.0, 26.4)	19.2 (2.4, 40.8)	0.146
ΔWeek 8–0	−2.4 (−14.4, 10.8)	2.4 (−1.2, 20.4)	0.0155
*p* value^‡^	0.2762	0.0464	

^†^Median, interquartile range (25%, 75%) in parentheses (all such values). ^‡^Within-group comparisons using Wilcoxon signed rank test. ^§^Between-group comparisons using Wilcoxon rank sum test.
